# Supplementary material for: Association of Endotoxemia with Low-Grade Inflammation, Metabolic Syndrome and Distinct Response to Lipopolysaccharide in Type 1 Diabetes
Source: Biomedicines. 2023 Dec 10;11(12):3269. doi: 10.3390/biomedicines11123269 (PMC10740930; doi:10.3390/biomedicines11123269)
Supplement: Supplementary file 1 [file biomedicines-11-03269-s001.zip › biomedicines-2658505-supplementary.pdf]

Supplementary materials for "Association of endotoxaemia with low grade inflammation, metabolic syndrome and distinct response to LPS in type 1 diabetes"

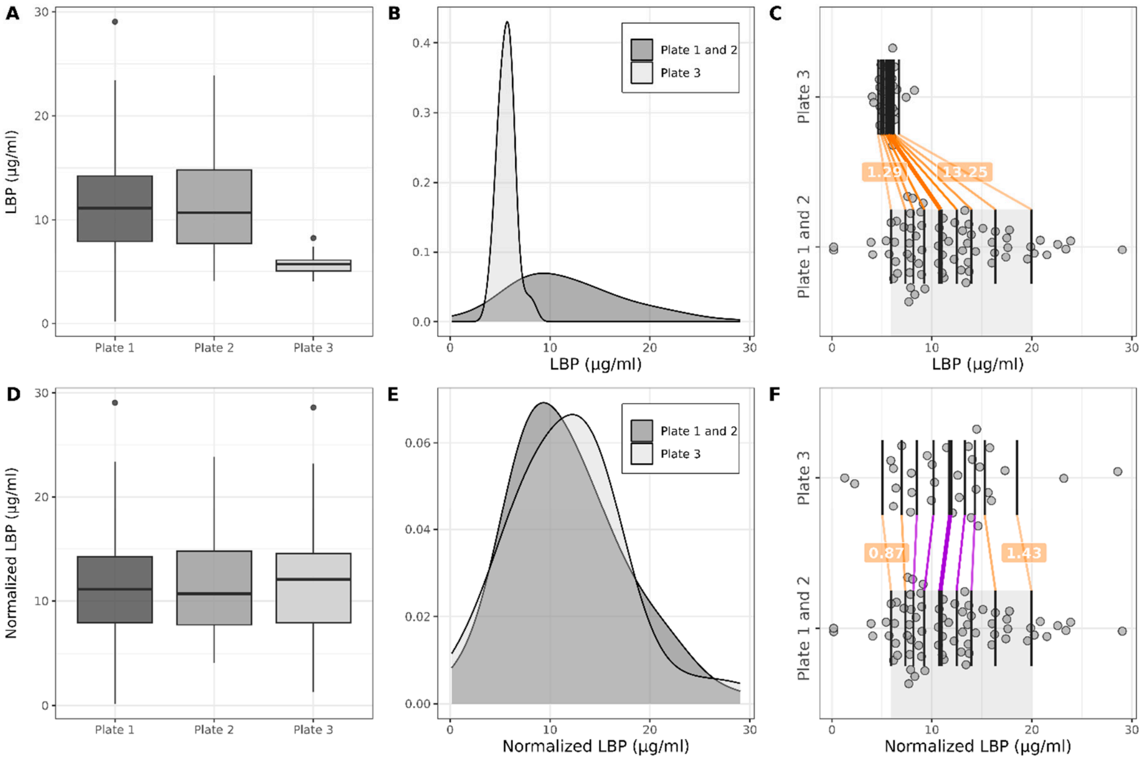

Supplemental Figure 1. Illustrations for LBP before and after the normalization. Boxplots by plates, density plots, and scatterplots with deciles for LBP raw data (upper panels) and after normalization using location-scale transformation between pooled plates 1 and 2 and plate 3 (lower panels), location parameter $\hat{\mu} = -24.83$, scale parameter $\hat{\sigma} = 6.475$ are used for transformation of plate 3.

Supplemental Table 1. Characteristics of subjects T1D and controls

Phenotype	Control (N=33)	T1D (N=74)	p
Male gender, N (%)	14 (42.4 %)	28 (37.8 %)	0.81
Age, years	35 (30 - 44)	43 (34 - 51)	0.013
BMI, kg/m^2	24.3 (22.3 - 27.9)	24.9 (22.6 - 28.4)	0.39
Waist/height ratio	0.47 (0.44 - 0.51)	0.49 (0.44 - 0.56)	0.16
Metabolic syndrome	4 (12.1 %)	31 (41.9 %)	0.005
Smokers, N (%)	9 (27.3 %)	20 (27.0 %)	1.00
Hypertension, N (%)	7 (21.2 %)	35 (47.3 %)	0.019
Length of diabetes, Years	-	21 (13 - 32)	-
Retinopathy, N (%)	-	32 (43.2 %)	-
CVD, N (%)	0 (0 %)	9 (12.2 %)	NS
On ACEI/ARB, N (%)	0 (0 %)	15 (20.3 %)	NS
On lipid lowering medication, N (%)	2 (6.1 %)	17 (23.0 %)	0.066
Autoimmune thyroid disease, N (%)	1 (3 %)	19 (25.7 %)	0.012
Hemoglobin A1C, %	5.2 (5.0 - 5.5)	7.7 (6.9 - 9.3)	<0.001
Hemoglobin A1C mmol/mol	31.4 (29.3 – 34.5)	56.5 (49.1 – 73.6)	<0.001
Estimated glomerular filtration rate, ml/min/1.73m^2	106 (94 - 113)	108 (91 - 117)	0.39
Albumin/creatinine ratio in urine, mg/mmol	0.32 (0.11 - 0.76)	0.57 (0.23 - 1.84)	0.013
Total cholesterol, mmol/l	4.7 (4.5 - 5.1)	5.0 (4.5 - 5.8)	0.14
Low density lipoproteins, mmol/l	2.8 (2.4 - 3.2)	3.0 (2.3 - 3.4)	0.77
High density lipoproteins, mmol/l	1.43 (1.27 - 1.61)	1.5 (1.3 - 1.9)	0.05
Triglycerides, mmol/l	0.98 (0.79 - 1.65)	1.2 (0.9 - 1.5)	0.21
Alanine aminotransaminase, U/l	21 (17 - 29)	19 (15 - 28)	0.18
Aspartate aminotransferase, U/l	25 (22 - 29)	22 (19 - 31)	0.13
Gamma-glutamyltransferase, U/l	16 (14 - 22)	16 (13 - 23)	0.93
Bilirubin, µmol/l	10.5 (7.1 – 15.0)	9.3 (7.0 – 12.0)	0.37
CRP, mg/l	0.5 (0.5 - 0.5)	0.9 (0.5 - 2.9)	<0.001
Haemoglobin, g/l	140 (127 - 152)	140 (132 - 149)	0.84

Erythrocytes, 10x12\L	4.6 (4.2 - 5.1)	4.7 (4.4 – 5.0)	0.62
Leukocytes, 10x9/L	6.2 (5.2 - 6.9)	6.2 (5.1 - 7.3)	0.90
Thrombocytes, 10x9/L	269 (225 - 301)	260 (230 - 288)	0.86
eGDR	7.1 (5.5 - 7.7)	3.3 (1.6 - 5.7)	<0.001
FLI	17.8 (6.6 - 34.6)	21.7 (9.9 - 48.2)	0.19
HSI	34.3 (30.8 - 40.2)	33.4 (29.9 - 39.5)	0.30

Supplemental Table 2. Comparisons of deciles of inflammatory markers in T1D patients stratified according to the presence of metabolic syndrome.

Variable	n	m	Decile 1 (10%)	Decile 2 (20%)	Decile 3 (30%)	Decile 4 (40%)	Decile 5 (50%)	Decile 6 (60%)	Decile 7 (70%)	Decile 8 (80%)	Decile 9 (90%)
**hsCRP, mg/l**	42	28	-0.09 (-0.28,0.15), p=0.44	0 (-0.24,0.33), p=0.971	0.09 (-0.22,0.53), p=0.595	0.2 (-0.21,0.76), p=0.371	0.34 (-0.22,1.01), p=0.25	0.48 (-0.22,1.34), p=0.182	0.62 (-0.29,1.67), p=0.159	0.78 (-1.24,2.49), p=0.414	0 (-2.14,2.83), p=0.998
**LPS, EU/ml**	42	29	0.01 (-0.03,0.05), p=0.78	0.03 (-0.01,0.08), p=0.193	0.05 (0,0.1), p=0.044	0.07 (0.02,0.13), p=0.01	0.09 (0.03,0.17), p=0.002	0.11 (0.04,0.21), p=0.002	0.15 (0.05,0.24), p=0.004	0.17 (0.05,0.26), p=0.013	0.16 (-0.02,0.27), p=0.091
**EndoCAb IgG, GMU/ml**	41	29	-11.74 (-30.66,7.65), p=0.232	-8.35 (-29.38,14.77), p=0.472	-5.21 (-27.25,19.49), p=0.666	-1.08 (-24.89,24.83), p=0.931	3.4 (-24.59,31.71), p=0.804	6.61 (-37.38,39.49), p=0.701	0.76 (-47.83,47.57), p=0.978	-8.9 (-55.47,50.36), p=0.72	-5.56 (-151.01,130.5), p=0.869
**EndoCAb IgM, MMU/ml**	42	28	1.37 (-8.72,11.15), p=0.791	-0.23 (-10.52,9.65), p=0.964	-1.78 (-12.92,8.83), p=0.738	-3.67 (-18.01,8.55), p=0.555	-6.77 (-25.8,7.96), p=0.391	-12.6 (-33.33,5.95), p=0.206	-19.83 (-41.22,1.08), p=0.063	-27.32 (-54.53,14.25), p=0.15	-25 (-141.55,75.6), p=0.281
**LBP, µg/ml**	42	29	2.45 (0.56,4.99), p=0.01	1.49 (-0.45,3.71), p=0.134	0.92 (-1.25,3.11), p=0.404	0.43 (-2.02,2.7), p=0.722	-0.14 (-2.9,2.28), p=0.914	-0.91 (-4.18,1.79), p=0.523	-2.02 (-5.84,1.34), p=0.233	-3.54 (-7.34,2.49), p=0.16	-3.64 (-9.13,4.79), p=0.395
**LPS/HDL ratio**	42	29	0.04 (0,0.08), p=0.079	0.05 (0,0.09), p=0.037	0.06 (0.01,0.1), p=0.018	0.06 (0.02,0.11), p=0.007	0.07 (0.02,0.13), p=0.003	0.08 (0.03,0.17), p=0.001	0.1 (0.03,0.3), p=0.002	0.19 (0.04,0.35), p=0.004	0.28 (0.09,0.48), p=0.004
**Calprotectin, µg/g**	35	27	-0.88 (-2.77,1.58), p=0.441	0.61 (-1.73,4.13), p=0.656	2.22 (-0.78,7.25), p=0.172	4.3 (0.23,9.98), p=0.036	6.9 (1.24,11.81), p=0.014	8.72 (2.47,13.13), p=0.011	9.39 (-13.65,13.98), p=0.18	5.35 (-18.29,14.99), p=1	-9.99 (-21.82,25.46), p=0.218

n – complete cases in T1D patients without metabolic syndrome; m – complete cases in T1D patients with metabolic syndrome.

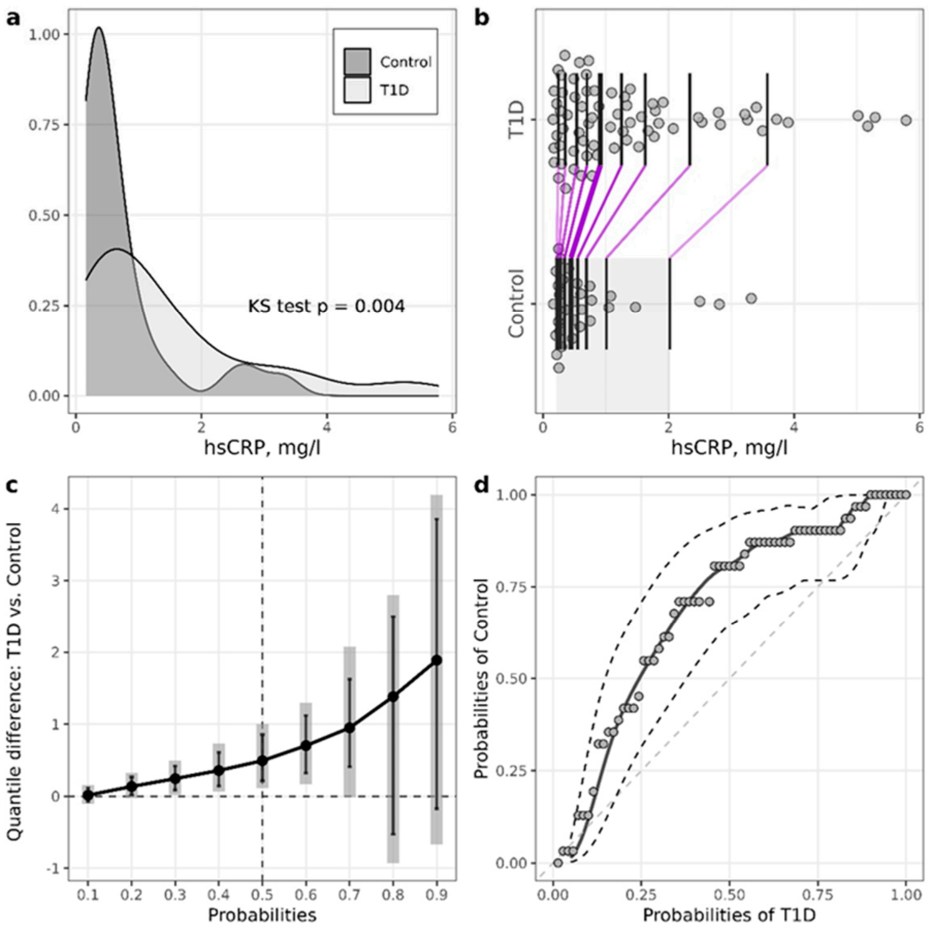

Figure 2. Illustrations for comparison of hsCRP levels between control and T1D groups. a – plots of densities in control and T1D, p-value of Kolmogorov-Smirnov (KS) test for the equality of distributions; b – one-dimensional scatterplots for control and T1D, vertical bars represents deciles of both groups (R library rogme); c – quantile difference (T1D - control) plot constructed at deciles, estimates obtained using two-sample smoothed empirical likelihood method (R library EL), error bars represent 95% pointwise CI's, shaded bars representing 95% simultaneous confidence bands for 9 deciles; d – probabilities of T1D versus probabilities of control (PP-plot) with estimate and 95% pointwise CI's obtained by two-sample smoothed empirical likelihood method (R library EL). Bandwidths for kernel estimation in c and d were selected using the method by Sheather and Jones (function bw.SJ in R).

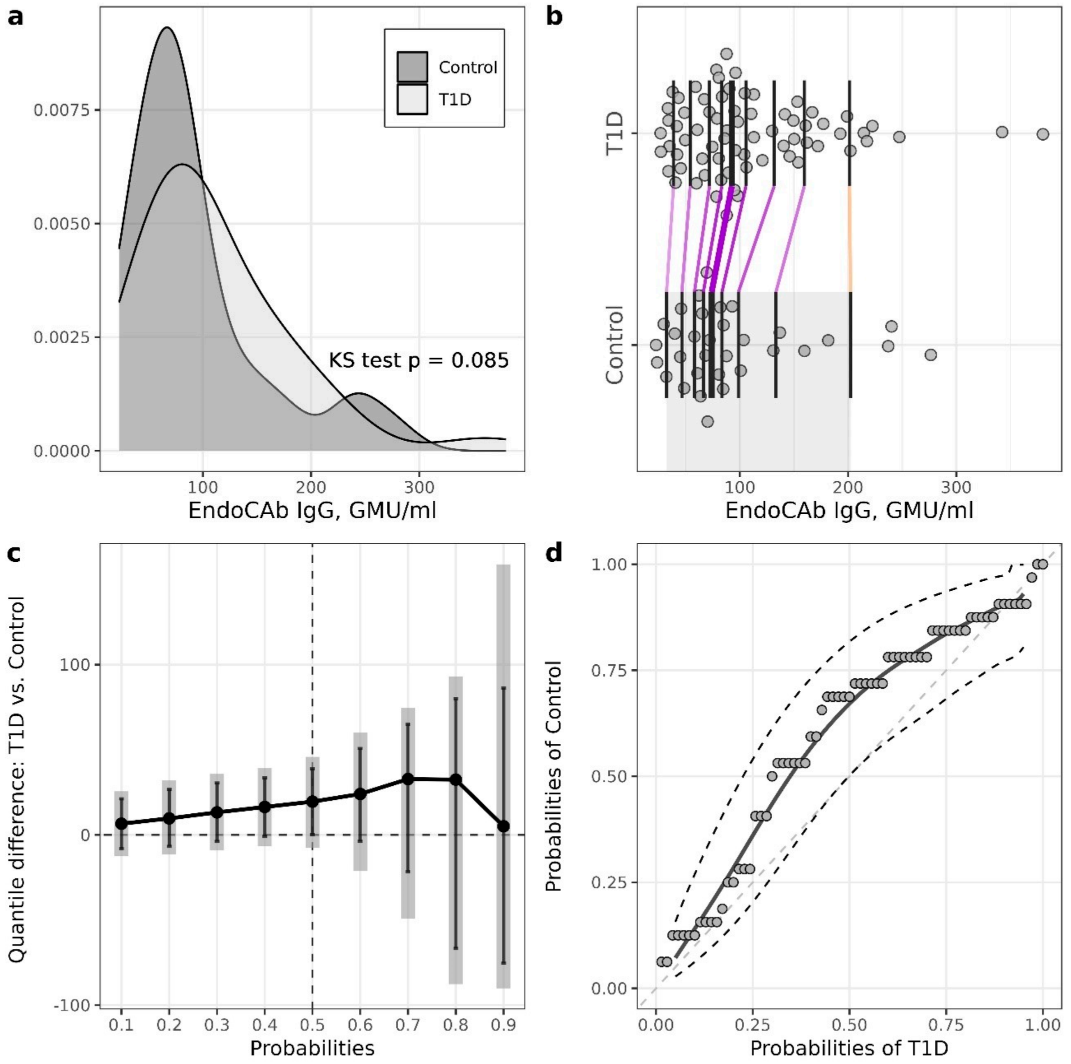

Supplemental Figure 3. Illustrations for comparison of EndoCAb IgG levels between control and T1D groups. a – plots of densities in control and T1D, p-value of Kolmogorov-Smirnov (KS) test for the equality of distributions; b – one-dimensional scatterplots for control and T1D, vertical bars represent deciles of both groups (R library rogme); c – quantile difference (T1D - control) plot constructed at deciles, estimates obtained using two-sample smoothed empirical likelihood method (R library EL), error bars represent 95% pointwise CI's, shaded bars representing 95% simultaneous confidence bands for 9 deciles; d – probabilities of T1D versus probabilities of control (PP-plot) with an estimate and 95% pointwise CI's obtained by two-sample smoothed empirical likelihood method (R library EL). Bandwidths for kernel estimation in c and d were selected using the method by Sheather and Jones (function bw.SJ in R).

Supplemental Table 3. Inflammatory marker analysis in patients with T1D stratified by metabolic syndrome.

Variable	No metabolic syndrome (N = 43)	Metabolic syndrome (N = 31)	P Wilcoxon	P ANCOVA	P Lepage	P KS test
**hsCRP, mg/l**	0.80 (0.49 - 1.53)	1.23 (0.38 - 2.17)	0.41	0.99	0.55	0.39
**LPS, EU/ml**	0.34 (0.30 - 0.40)	0.42 (0.35 - 0.56)	0.009	0.020	0.018	0.009
**EndoCAb IgG, GMU/ml**	89.5 (67.1 - 150.2)	96.0 (59.1 - 141.6)	0.84	0.54	0.88	0.85
**Endo CAb IgM, MMU/ml**	46.6 (34.2 - 85.2)	43.7 (31.3 - 60.4)	0.33	0.31	0.24	0.18
**LBP, µg/ml**	11.1 (7.9 - 16.3)	11.1 (7.9 - 13.3)	0.89	0.66	0.20	0.54
**LPS/HDL**	0.22 (0.17 - 0.26)	0.28 (0.24 - 0.38)	0.001	<0.001	0.004	0.003
**Calprotectin, µg/g**	3.8 (2.7 - 8.6)	12.5 (3.2 - 18.4)	0.18	0.23	0.40	0.057

KS – Kolmogorov-Smirnov test for equality of two distributions. ANCOVA on rank-transformed data with covariates: sex, diabetes duration, age, and BMI

Supplemental Table 4. Markers of endotoxaemia and inflammation in the study groups.

Phenotype	**Control**		**Type 1 diabetes**		**P**			**Difference of deciles: T1D - Control**								
	Median (IQR)	N	Median (IQR)	N	Wilcoxon	ANCOVA	Lepage	10%	20%	30%	40%	50%	60%	70%	80%	90%
hsCRP, mg/l	0.47 (0.26 - 0.75)	31	0.84 (0.49 - 1.78)	70	0.002	0.042	0.008	0.01 (-0.07, 0.11), p=0.76	0.13 (0.02, 0.27), p=0.024	0.24 (0.09, 0.42), p=0.004	0.36 (0.14, 0.61), p=0.001	0.49 (0.21, 0.86), p=0.001	0.7 (0.32, 1.12), p=0.002	0.95 (0.41, 1.63), p=0.008	1.38 (-0.53, 2.5), p=0.12	1.89 (-0.17, 3.86), p=0.08
LPS, EU/ml	0.39 (0.31 – 0.49)	31	0.36 (0.31 - 0.45)	71	0.49	0.25	0.74	0.01 (-0.02, 0.04), p=0.47	0 (-0.04, 0.04), p=0.99	-0.01 (-0.06, 0.03), p=0.64	-0.02 (-0.07, 0.03), p=0.44	-0.03 (-0.09, 0.03), p=0.34	-0.03 (-0.12, 0.03), p=0.31	-0.04 (-0.16, 0.05), p=0.33	-0.05 (-0.19, 0.09), p=0.48	-0.04 (-0.17, 0.08), p=0.55
EndoCAb IgG, GMU/ml	71.1 (55.7 - 101.5)	32	92.6 (65.8 - 148.6)	70	0.091	0.32	0.23	6.55 (-8.1, 21.18), p=0.39	9.67 (-6.78, 26.8), p=0.26	13.22 (-3.88, 30.46), p=0.13	16.4 (-0.9, 33.6), p=0.063	19.5 (0.2, 38.8), p=0.048	24.0 (-3.8, 50.7), p=0.075	32.8 (-21.6, 65.1), p=0.17	32.5 (-66.6, 79.9), p=0.37	5.1 (-75.2, 86.2), p=0.95

EndoCAb IgM, MMU/ml	48.3 (27.4 - 69.6)	32	45.8 (33.4 - 69.5)	70	0.64	0.093	0.60	1.71 (-5.66, 8.62), p=0.64	3.02 (-5.54, 10.31), p=0.46	3.26 (-7.68, 11.71), p=0.51	2.65 (-10.82, 13.5), p=0.68	1.58 (-13.71, 16.02), p=0.84	1.62 (-19.48, 18.49), p=0.85	2.22 (-33.0, 22.8), p=0.85	-4.34 (-46.67, 31.85), p=0.85	-9.44 (-74.85, -37.13), p=0.69
LBP, µg/ml	10.1 (7.0 - 14.2)	32	11.1 (7.9 - 14.0)	71	0.60	0.84	0.72	0.49 (-1.7, 4.81), p=0.64	0.67 (-1.13, 2.59), p=0.44	0.73 (-1.41, 2.62), p=0.48	0.77 (-2.64, 2.99), p=0.54	0.53 (-2.84, 3.18), p=0.81	-0.42 (-2.45, 3.22), p=0.78	-0.03 (-1.89, 2.73), p=0.98	1.6 (-1.07, 5.37), p=0.29	4.84 (1.39, 7.94), p=0.006
LPS/HDL ratio	0.24 (0.20 – 0.36)	31	0.24 (0.19 – 0.30)	71	0.27	0.087	0.48	-0.04 (-0.06, -0.01), p=0.018	-0.02 (-0.05, 0.01), p=0.11	-0.01 (-0.05, 0.02), p=0.46	0 (-0.07, 0.03), p=0.82	-0.01 (-0.09, 0.03), p=0.81	-0.04 (-0.13, 0.04), p=0.45	-0.05 (-0.25, 0.03), p=0.15	-0.18 (-0.26, 0.03), p=0.19	-0.13 (-0.39, 0.06), p=0.23
Calprotectin, µg/g	6.4 (3.9 – 10.3)	30	5.8 (2.7 – 16.2)	62	0.59	0.56	0.002	-2.14 (-3.34, -0.44), p=0.017	-1.86 (-3.26, -0.40), p=0.016	-1.54 (-3.21, 0.14), p=0.071	-1.12 (-3.2, 1.11), p=0.30	-0.47 (-3.18, 2.81), p=0.74	0.7 (-3.0, 7.4), p=0.71	4.7 (-2.1, 10.7), p=0.24	8.0 (-1.8, 17.9), p=0.083	13.9 (-3.6, 29.0), p=0.24

ANCOVA on rank-transformed data with covariates: sex, diabetes duration, age, and BMI

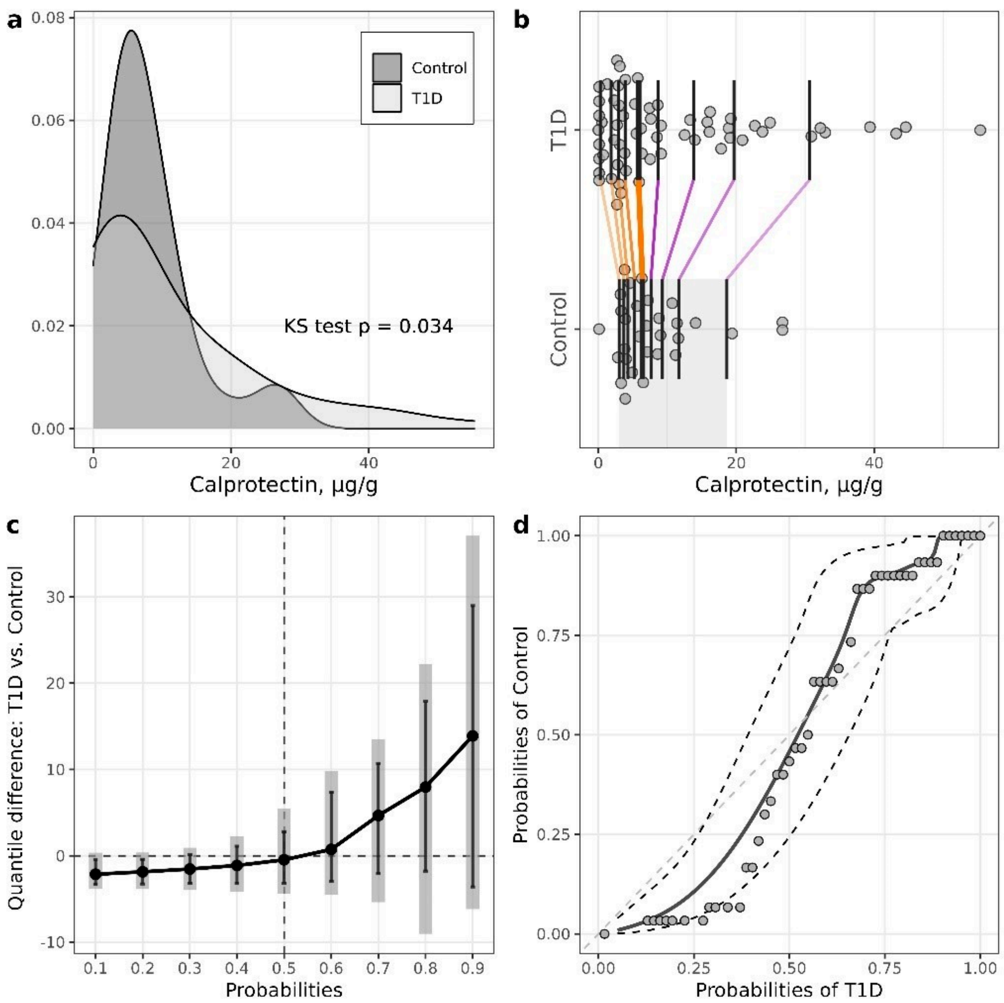

Supplemental Figure 4. Illustrations for comparison of calprotectin levels between control and T1D groups. a – plots of densities in control and T1D, a p-value of Kolmogorov-Smirnov (KS) test for the equality of distributions; b – one-dimensional scatterplots for control and T1D, vertical bars represents deciles of both groups (R library rogme); c – quantile difference (T1D - control) plot constructed at deciles, estimates obtained using two-sample smoothed empirical likelihood method (R library EL), error bars represent 95% pointwise CI's, shaded bars representing 95% simultaneous confidence bands for 9 deciles; d – probabilities of T1D versus probabilities of control (PP-plot) with estimate and 95% pointwise CI's obtained by two-sample smoothed empirical likelihood method (R library EL). Bandwidths for kernel estimation in c and d were selected using the method by Sheather and Jones (function bw. SJ in R).

Supplemental Table 5. Correlations in the type 1 diabetes group. Spearman correlation coefficient (95% CI) was calculated.

Variable	CRO, mg/l	hsCRP, mg/l	LPS, EU/ml	endoCAb IgG, GMU/ml	endoCAb IgM, MMU/ml	LBP, µg/ml	LPS/HDL	Calprotectin, µg/g
**Serum glucose**	0.03, (-0.2, 0.26), p=0.786	0.12, (-0.12, 0.34), p=0.337	0.04, (-0.2, 0.27), p=0.742	0, (-0.23, 0.24), p=0.976	-0.08, (-0.31, 0.16), p=0.512	0.12, (-0.12, 0.34), p=0.336	0.19, (-0.05, 0.41), p=0.109	0.08, (-0.16, 0.32), p=0.496
**HbA1c**	0.03, (-0.2, 0.25), p=0.825	0.11, (-0.13, 0.34), p=0.368	-0.03, (-0.26, 0.21), p=0.833	0.27, (0.03, 0.47), p=0.026	0.11, (-0.13, 0.34), p=0.366	0.05, (-0.19, 0.28), p=0.707	0.05, (-0.19, 0.28), p=0.706	0.03, (-0.21, 0.27), p=0.783
**eGFR**	-0.24, (-0.45, -0.01), p=0.038	-0.02, (-0.26, 0.21), p=0.861	-0.06, (-0.29, 0.18), p=0.614	-0.1, (-0.33, 0.13), p=0.391	-0.21, (-0.43, 0.03), p=0.079	0.07, (-0.17, 0.29), p=0.584	0.08, (-0.16, 0.31), p=0.5	0.11, (-0.13, 0.34), p=0.377
**Albumin/creatinine ratio in urine**	0.13, (-0.11, 0.35), p=0.277	0.12, (-0.13, 0.35), p=0.342	-0.19, (-0.41, 0.06), p=0.128	-0.03, (-0.27, 0.21), p=0.809	-0.07, (-0.31, 0.17), p=0.544	-0.03, (-0.26, 0.21), p=0.807	-0.11, (-0.34, 0.13), p=0.352	0.1, (-0.15, 0.33), p=0.436
**Weight**	0.38, (0.16, 0.57), p=0.001	0.07, (-0.17, 0.3), p=0.568	0.04, (-0.2, 0.27), p=0.751	-0.08, (-0.31, 0.16), p=0.496	0.02, (-0.21, 0.25), p=0.862	-0.01, (-0.25, 0.22), p=0.916	0.06, (-0.18, 0.29), p=0.617	-0.07, (-0.3, 0.18), p=0.595
**Body mass index**	0.55, (0.35, 0.7), p=0	0.17, (-0.07, 0.39), p=0.155	0.14, (-0.09, 0.37), p=0.23	0.01, (-0.22, 0.25), p=0.917	0.01, (-0.22, 0.25), p=0.918	0.05, (-0.19, 0.28), p=0.697	0.1, (-0.14, 0.33), p=0.412	-0.01, (-0.25, 0.23), p=0.916
**Waist/height ratio**	0.47, (0.26, 0.64), p=0	0.23, (-0.01, 0.44), p=0.057	0.14, (-0.1, 0.36), p=0.243	-0.08, (-0.31, 0.16), p=0.508	-0.11, (-0.34, 0.12), p=0.345	0.03, (-0.2, 0.26), p=0.781	0.16, (-0.08, 0.38), p=0.193	-0.09, (-0.32, 0.15), p=0.473
**eGDR**	-0.26, (-0.46, -0.03), p=0.027	-0.28, (-0.48, -0.04), p=0.02	0.01, (-0.23, 0.24), p=0.967	-0.05, (-0.28, 0.18), p=0.664	0.07, (-0.17, 0.3), p=0.572	0.06, (-0.18, 0.29), p=0.632	-0.06, (-0.29, 0.18), p=0.63	0.04, (-0.2, 0.28), p=0.743
**HSI**	0.52, (0.31, 0.68), p=0	0.21, (-0.03, 0.43), p=0.088	0.13, (-0.11, 0.36), p=0.283	0.04, (-0.21, 0.27), p=0.77	0.04, (-0.2, 0.28), p=0.728	-0.03, (-0.26, 0.21), p=0.832	0.14, (-0.1, 0.37), p=0.25	-0.06, (-0.3, 0.19), p=0.624
**FLI**	0.48, (0.27, 0.64), p=0	0.22, (-0.02, 0.43), p=0.07	0.24, (0.01, 0.45), p=0.04	-0.08, (-0.31, 0.16), p=0.514	-0.06, (-0.29, 0.17), p=0.603	0.06, (-0.18, 0.29), p=0.63	0.23, (-0.01, 0.44), p=0.053	-0.06, (-0.29, 0.18), p=0.641
**ALT**	0.02, (-0.21, 0.25), p=0.859	-0.04, (-0.27, 0.2), p=0.742	-0.07, (-0.3, 0.17), p=0.575	-0.09, (-0.32, 0.15), p=0.445	-0.07, (-0.3, 0.16), p=0.543	-0.07, (-0.29, 0.17), p=0.585	-0.04, (-0.27, 0.19), p=0.726	-0.02, (-0.26, 0.22), p=0.883
**AST**	0, (-0.23, 0.23), p=0.996	-0.07, (-0.31, 0.17), p=0.568	-0.1, (-0.33, 0.14), p=0.424	0, (-0.24, 0.24), p=0.976	-0.14, (-0.37, 0.1), p=0.257	0.03, (-0.21, 0.27), p=0.789	-0.19, (-0.41, 0.05), p=0.115	0.07, (-0.17, 0.32), p=0.556
**GGT**	0.14, (-0.09, 0.36), p=0.239	0.05, (-0.19, 0.28), p=0.689	-0.04, (-0.27, 0.2), p=0.771	-0.21, (-0.43, 0.03), p=0.076	-0.06, (-0.29, 0.17), p=0.6	-0.01, (-0.24, 0.23), p=0.954	-0.07, (-0.3, 0.17), p=0.568	-0.05, (-0.29, 0.19), p=0.659
**Transglutaminase IgA**	-0.04, (-0.27, 0.19), p=0.738	-0.21, (-0.43, 0.03), p=0.081	0.07, (-0.16, 0.3), p=0.541	0.01, (-0.22, 0.25), p=0.913	0.11, (-0.13, 0.34), p=0.379	0.04, (-0.19, 0.28), p=0.717	0.05, (-0.18, 0.28), p=0.666	-0.28, (-0.49, -0.04), p=0.022
**Total bilirubin**	-0.18, (-0.4, 0.05), p=0.117	-0.16, (-0.38, 0.08), p=0.182	0, (-0.24, 0.23), p=0.968	-0.1, (-0.33, 0.14), p=0.399	0.03, (-0.2, 0.27), p=0.779	0, (-0.24, 0.23), p=0.967	-0.06, (-0.28, 0.18), p=0.648	-0.14, (-0.37, 0.1), p=0.251
**Erythrocytes**	-0.03, (-0.25, 0.2), p=0.816	0, (-0.24, 0.23), p=0.991	0.2, (-0.03, 0.42), p=0.091	-0.04, (-0.27, 0.2), p=0.765	0.1, (-0.14, 0.33), p=0.393	-0.01, (-0.24, 0.22), p=0.92	0.26, (0.02, 0.47), p=0.03	0.09, (-0.16, 0.32), p=0.494
**Hemoglobin**	-0.15, (-0.37, 0.08), p=0.207	-0.06, (-0.29, 0.18), p=0.623	0.18, (-0.06, 0.4), p=0.14	-0.2, (-0.42, 0.04), p=0.1	0.04, (-0.19, 0.28), p=0.715	-0.07, (-0.3, 0.16), p=0.549	0.33, (0.1, 0.53), p=0.005	0.03, (-0.21, 0.27), p=0.792
**Leukocytes**	0.1, (-0.13, 0.32), p=0.391	0.18, (-0.06, 0.4), p=0.13	-0.06, (-0.29, 0.18), p=0.62	-0.07, (-0.3, 0.17), p=0.589	-0.06, (-0.29, 0.17), p=0.605	0.15, (-0.09, 0.37), p=0.211	0.04, (-0.19, 0.27), p=0.723	-0.07, (-0.3, 0.18), p=0.588
**Total cholesterol**	0.29, (0.06, 0.49), p=0.013	0.13, (-0.11, 0.36), p=0.276	0.04, (-0.2, 0.27), p=0.746	0.18, (-0.06, 0.4), p=0.137	0.07, (-0.17, 0.3), p=0.555	0.2, (-0.04, 0.42), p=0.092	-0.18, (-0.39, 0.06), p=0.142	0.14, (-0.11, 0.37), p=0.263
**High-density cholesterol**	0.16, (-0.07, 0.38), p=0.171	0.13, (-0.1, 0.36), p=0.267	-0.17, (-0.39, 0.06), p=0.146	0.32, (0.09, 0.52), p=0.006	0.26, (0.02, 0.47), p=0.028	0.12, (-0.12, 0.34), p=0.324	-0.7, (-0.81, -0.54), p=0	-0.06, (-0.3, 0.18), p=0.614
**Low-density cholesterol**	0.21, (-0.02, 0.42), p=0.075	0.04, (-0.19, 0.28), p=0.723	-0.08, (-0.31, 0.15), p=0.48	0.04, (-0.19, 0.27), p=0.729	-0.05, (-0.28, 0.19), p=0.683	0.17, (-0.07, 0.39), p=0.164	-0.01, (-0.24, 0.23), p=0.95	0.22, (-0.03, 0.44), p=0.08
**Tryglicerides**	0.12, (-0.11, 0.34), p=0.312	0.13, (-0.11, 0.36), p=0.282	0.49, (0.28, 0.66), p=0	-0.08, (-0.31, 0.16), p=0.537	0, (-0.23, 0.24), p=0.982	-0.01, (-0.24, 0.22), p=0.929	0.52, (0.31, 0.68), p=0	0.04, (-0.21, 0.27), p=0.776

**Diabetes duration**	0.14, (-0.09, 0.36), p=0.224	0.15, (-0.09, 0.37), p=0.226	-0.09, (-0.32, 0.15), p=0.463	-0.07, (-0.3, 0.17), p=0.576	0.11, (-0.13, 0.34), p=0.354	-0.09, (-0.31, 0.15), p=0.478	-0.22, (-0.43, 0.02), p=0.069	0.02, (-0.22, 0.26), p=0.845
**CRP**	-	0.61, (0.42, 0.75), p=0	0.13, (-0.11, 0.35), p=0.296	0.16, (-0.08, 0.38), p=0.192	0.01, (-0.23, 0.24), p=0.951	0.18, (-0.06, 0.4), p=0.137	-0.04, (-0.27, 0.2), p=0.767	-0.16, (-0.39, 0.09), p=0.197
**hsCRP**	0.61, (0.42, 0.75), p=0	-	0.18, (-0.06, 0.4), p=0.14	0.22, (-0.02, 0.44), p=0.07	0, (-0.24, 0.23), p=0.972	0.36, (0.13, 0.55), p=0.002	0.01, (-0.23, 0.24), p=0.932	-0.15, (-0.38, 0.11), p=0.256
**LPS**	0.13, (-0.11, 0.35), p=0.296	0.18, (-0.06, 0.4), p=0.14	-	0.07, (-0.17, 0.3), p=0.566	0.13, (-0.11, 0.36), p=0.268	0.04, (-0.2, 0.27), p=0.771	0.78, (0.64, 0.86), p=0	-0.02, (-0.27, 0.22), p=0.853
**EndoCAb IgG**	0.16, (-0.08, 0.38), p=0.192	0.22, (-0.02, 0.44), p=0.07	0.07, (-0.17, 0.3), p=0.566	-	0.23, (-0.01, 0.45), p=0.052	0.23, (-0.01, 0.45), p=0.054	-0.14, (-0.36, 0.1), p=0.258	0.13, (-0.12, 0.37), p=0.296
**EndoCAb IgM**	0.01, (-0.23, 0.24), p=0.951	0, (-0.24, 0.23), p=0.972	0.13, (-0.11, 0.36), p=0.268	0.23, (-0.01, 0.45), p=0.052	-	-0.14, (-0.36, 0.1), p=0.263	-0.14, (-0.37, 0.09), p=0.231	-0.05, (-0.29, 0.2), p=0.695
**LBP**	0.18, (-0.06, 0.4), p=0.137	0.36, (0.13, 0.55), p=0.002	0.04, (-0.2, 0.27), p=0.771	0.23, (-0.01, 0.45), p=0.054	-0.14, (-0.36, 0.1), p=0.263	-	-0.02, (-0.25, 0.21), p=0.854	-0.05, (-0.29, 0.2), p=0.721
**LPS/HDL**	-0.04, (-0.27, 0.2), p=0.767	0.01, (-0.23, 0.24), p=0.932	0.78, (0.64, 0.86), p=0	-0.14, (-0.36, 0.1), p=0.258	-0.14, (-0.37, 0.09), p=0.231	-0.02, (-0.25, 0.21), p=0.854	-	-0.04, (-0.29, 0.2), p=0.733
**Calprotectin**	-0.16, (-0.39, 0.09), p=0.197	-0.15, (-0.38, 0.11), p=0.256	-0.02, (-0.27, 0.22), p=0.853	0.13, (-0.12, 0.37), p=0.296	-0.05, (-0.29, 0.2), p=0.695	-0.05, (-0.29, 0.2), p=0.721	-0.04, (-0.29, 0.2), p=0.733	-

Supplemental **Table 6.** Correlations in the control group. Spearman correlation coefficient (95% CI) was calculated.

Variable	CRO, mg/l	hsCRP, mg/l	LPS, EU/ml	endoCAb IgG, GMU/ml	endoCAb IgM, MMU/ml	LBP, µg/ml	LPS/HDL	Calprotectin, µg/g
**Serum glucose**	0.2, (-0.15, 0.51), p=0.256	-0.05, (-0.4, 0.31), p=0.778	-0.13, (-0.46, 0.23), p=0.48	-0.28, (-0.58, 0.08), p=0.119	-0.24, (-0.55, 0.12), p=0.183	0.17, (-0.19, 0.49), p=0.343	-0.21, (-0.52, 0.16), p=0.255	-0.14, (-0.48, 0.23), p=0.449
**HbA1c**	0.34, (-0.02, 0.62), p=0.056	0.5, (0.16, 0.74), p=0.004	-0.13, (-0.46, 0.23), p=0.483	0.15, (-0.21, 0.48), p=0.404	0.24, (-0.12, 0.55), p=0.183	0.18, (-0.18, 0.5), p=0.318	-0.13, (-0.46, 0.23), p=0.487	0.17, (-0.21, 0.5), p=0.373
**eGFR**	-0.22, (-0.53, 0.13), p=0.208	-0.21, (-0.53, 0.16), p=0.249	-0.11, (-0.44, 0.25), p=0.538	0.09, (-0.27, 0.43), p=0.619	0.06, (-0.29, 0.4), p=0.737	-0.21, (-0.52, 0.15), p=0.248	-0.09, (-0.43, 0.27), p=0.624	-0.06, (-0.41, 0.31), p=0.772
**Albumin/creatinine ratio in urine**	-0.26, (-0.56, 0.09), p=0.138	-0.32, (-0.61, 0.05), p=0.081	0.02, (-0.33, 0.36), p=0.925	0.1, (-0.26, 0.44), p=0.578	-0.19, (-0.51, 0.17), p=0.287	-0.03, (-0.38, 0.32), p=0.865	-0.02, (-0.37, 0.33), p=0.898	0.2, (-0.18, 0.52), p=0.298
**Weight**	0.39, (0.04, 0.65), p=0.026	0.19, (-0.18, 0.52), p=0.296	0.23, (-0.14, 0.54), p=0.211	0.04, (-0.31, 0.39), p=0.811	-0.44, (-0.69, -0.09), p=0.012	0.12, (-0.24, 0.45), p=0.498	0.35, (-0.01, 0.63), p=0.05	-0.02, (-0.38, 0.34), p=0.904
**Body mass index**	0.42, (0.07, 0.67), p=0.016	0.38, (0.02, 0.66), p=0.034	0.13, (-0.23, 0.46), p=0.476	0.08, (-0.27, 0.42), p=0.654	-0.15, (-0.48, 0.21), p=0.405	0.16, (-0.2, 0.48), p=0.387	0.27, (-0.09, 0.57), p=0.136	-0.1, (-0.45, 0.27), p=0.586
**Waist/height ratio**	0.37, (0.02, 0.64), p=0.034	0.33, (-0.04, 0.62), p=0.074	0.19, (-0.18, 0.5), p=0.309	0.08, (-0.28, 0.42), p=0.661	-0.2, (-0.52, 0.16), p=0.267	0.27, (-0.1, 0.57), p=0.141	0.23, (-0.14, 0.54), p=0.21	-0.15, (-0.49, 0.22), p=0.422
**eGDR**	-0.34, (-0.62, 0.01), p=0.051	-0.26, (-0.57, 0.11), p=0.16	-0.25, (-0.56, 0.11), p=0.16	-0.1, (-0.44, 0.26), p=0.574	0.14, (-0.22, 0.47), p=0.444	-0.13, (-0.46, 0.23), p=0.479	-0.32, (-0.61, 0.04), p=0.077	-0.12, (-0.46, 0.25), p=0.534
**HSI**	0.46, (0.12, 0.7), p=0.007	0.43, (0.07, 0.69), p=0.016	0.09, (-0.26, 0.43), p=0.605	0.07, (-0.28, 0.41), p=0.697	-0.08, (-0.42, 0.28), p=0.671	0.14, (-0.22, 0.46), p=0.456	0.24, (-0.12, 0.55), p=0.178	-0.2, (-0.52, 0.18), p=0.3
**FLI**	0.38, (0.02, 0.64), p=0.031	0.24, (-0.13, 0.55), p=0.198	0.49, (0.16, 0.73), p=0.004	0.01, (-0.34, 0.36), p=0.958	-0.42, (-0.68, -0.07), p=0.017	0.27, (-0.1, 0.57), p=0.14	0.54, (0.21, 0.76), p=0.002	0.01, (-0.35, 0.37), p=0.953
**ALT**	0.49, (0.16, 0.73), p=0.003	0.32, (-0.04, 0.61), p=0.076	0.12, (-0.24, 0.45), p=0.524	0.06, (-0.3, 0.4), p=0.764	-0.1, (-0.43, 0.26), p=0.605	0.23, (-0.14, 0.54), p=0.215	0.31, (-0.06, 0.6), p=0.087	-0.13, (-0.47, 0.24), p=0.48
**AST**	0.31, (-0.05, 0.59), p=0.081	-0.02, (-0.38, 0.33), p=0.895	0, (-0.35, 0.35), p=0.986	0.33, (-0.03, 0.62), p=0.063	-0.04, (-0.39, 0.31), p=0.819	0.18, (-0.18, 0.5), p=0.311	0.13, (-0.23, 0.46), p=0.493	-0.13, (-0.47, 0.25), p=0.506

**GGT**	0.36, (0.01, 0.64), p=0.037	0.29, (-0.08, 0.59), p=0.119	0.16, (-0.2, 0.49), p=0.368	0.41, (0.05, 0.67), p=0.021	0.13, (-0.23, 0.46), p=0.486	0.21, (-0.15, 0.53), p=0.238	0.3, (-0.06, 0.59), p=0.097	-0.02, (-0.38, 0.34), p=0.901
**Transglutaminase IgA**	-0.4, (-0.66, -0.05), p=0.021	-0.33, (-0.62, 0.04), p=0.07	-0.11, (-0.44, 0.25), p=0.557	0.22, (-0.15, 0.53), p=0.235	0.07, (-0.28, 0.41), p=0.696	-0.12, (-0.45, 0.24), p=0.518	-0.02, (-0.37, 0.33), p=0.907	0.37, (0, 0.65), p=0.041
**Total bilirubin**	-0.24, (-0.55, 0.11), p=0.173	0.04, (-0.32, 0.39), p=0.84	0.09, (-0.26, 0.43), p=0.609	0.35, (-0.01, 0.63), p=0.052	0.03, (-0.32, 0.38), p=0.863	-0.13, (-0.46, 0.23), p=0.484	-0.02, (-0.36, 0.33), p=0.921	-0.19, (-0.52, 0.19), p=0.318
**Erythrocytes**	0.12, (-0.24, 0.44), p=0.511	0.02, (-0.33, 0.38), p=0.899	0.45, (0.1, 0.7), p=0.01	0.03, (-0.32, 0.38), p=0.855	-0.37, (-0.64, -0.01), p=0.039	0.31, (-0.05, 0.6), p=0.084	0.5, (0.16, 0.73), p=0.004	0.17, (-0.2, 0.51), p=0.355
**Hemoglobin**	-0.04, (-0.38, 0.31), p=0.839	-0.19, (-0.51, 0.18), p=0.312	0.43, (0.08, 0.69), p=0.014	-0.1, (-0.44, 0.26), p=0.579	-0.4, (-0.67, -0.05), p=0.022	0.32, (-0.04, 0.61), p=0.074	0.51, (0.18, 0.74), p=0.003	0.21, (-0.16, 0.53), p=0.26
**Leukocytes**	-0.2, (-0.51, 0.16), p=0.276	-0.09, (-0.43, 0.27), p=0.614	0.32, (-0.04, 0.61), p=0.075	-0.2, (-0.52, 0.16), p=0.273	-0.05, (-0.4, 0.3), p=0.77	0.34, (-0.02, 0.62), p=0.057	0.33, (-0.03, 0.62), p=0.062	0.3, (-0.07, 0.6), p=0.103
**Total cholesterol**	-0.06, (-0.39, 0.29), p=0.746	-0.23, (-0.54, 0.14), p=0.221	0.07, (-0.29, 0.41), p=0.721	-0.12, (-0.45, 0.24), p=0.515	-0.17, (-0.5, 0.19), p=0.339	0.01, (-0.34, 0.36), p=0.95	-0.12, (-0.45, 0.24), p=0.505	-0.1, (-0.44, 0.27), p=0.615
**High-density cholesterol**	-0.24, (-0.55, 0.11), p=0.17	-0.15, (-0.48, 0.21), p=0.409	-0.32, (-0.61, 0.04), p=0.073	-0.19, (-0.51, 0.17), p=0.302	0.18, (-0.19, 0.5), p=0.336	-0.21, (-0.52, 0.15), p=0.245	-0.68, (-0.84, -0.4), p=0	-0.27, (-0.58, 0.1), p=0.144
**Low-density cholesterol**	-0.09, (-0.42, 0.27), p=0.637	-0.21, (-0.52, 0.16), p=0.267	-0.04, (-0.38, 0.31), p=0.836	-0.05, (-0.4, 0.3), p=0.77	-0.18, (-0.5, 0.18), p=0.322	-0.01, (-0.36, 0.34), p=0.951	-0.03, (-0.37, 0.32), p=0.872	0.16, (-0.21, 0.49), p=0.395
**Tryglicerides**	0.28, (-0.07, 0.57), p=0.112	0.06, (-0.31, 0.4), p=0.767	0.79, (0.57, 0.9), p=0	-0.09, (-0.43, 0.27), p=0.614	-0.6, (-0.8, -0.29), p=0	0.28, (-0.08, 0.58), p=0.118	0.67, (0.39, 0.84), p=0	0.17, (-0.2, 0.5), p=0.363
**CRP**	-	0.42, (0.06, 0.68), p=0.018	0.05, (-0.31, 0.39), p=0.796	-0.02, (-0.37, 0.33), p=0.895	-0.01, (-0.35, 0.34), p=0.973	-0.02, (-0.36, 0.33), p=0.92	0.14, (-0.22, 0.46), p=0.455	-0.22, (-0.54, 0.16), p=0.241
**hsCRP**	0.42, (0.06, 0.68), p=0.018	-	0.23, (-0.14, 0.54), p=0.217	0.1, (-0.26, 0.44), p=0.584	0.19, (-0.18, 0.51), p=0.308	0.21, (-0.16, 0.53), p=0.254	0.2, (-0.17, 0.52), p=0.269	-0.22, (-0.55, 0.17), p=0.252
**LPS**	0.05, (-0.31, 0.39), p=0.796	0.23, (-0.14, 0.54), p=0.217	-	-0.09, (-0.43, 0.27), p=0.627	-0.41, (-0.67, -0.06), p=0.021	0.36, (0, 0.64), p=0.045	0.86, (0.71, 0.94), p=0	0.15, (-0.23, 0.49), p=0.432
**EndoCab IgG**	-0.02, (-0.37, 0.33), p=0.895	0.1, (-0.26, 0.44), p=0.584	-0.09, (-0.43, 0.27), p=0.627	-	0.11, (-0.25, 0.44), p=0.565	-0.29, (-0.59, 0.07), p=0.103	-0.02, (-0.37, 0.33), p=0.897	0, (-0.36, 0.37), p=0.984
**EndoCab IgM**	-0.01, (-0.35, 0.34), p=0.973	0.19, (-0.18, 0.51), p=0.308	-0.41, (-0.67, -0.06), p=0.021	0.11, (-0.25, 0.44), p=0.565	-	-0.05, (-0.39, 0.3), p=0.775	-0.28, (-0.58, 0.08), p=0.117	-0.21, (-0.54, 0.17), p=0.267
**LBP**	-0.02, (-0.36, 0.33), p=0.92	0.21, (-0.16, 0.53), p=0.254	0.36, (0, 0.64), p=0.045	-0.29, (-0.59, 0.07), p=0.103	-0.05, (-0.39, 0.3), p=0.775	-	0.39, (0.03, 0.66), p=0.03	-0.08, (-0.44, 0.29), p=0.673
**LPS/HDL**	0.14, (-0.22, 0.46), p=0.455	0.2, (-0.17, 0.52), p=0.269	0.86, (0.71, 0.94), p=0	-0.02, (-0.37, 0.33), p=0.897	-0.28, (-0.58, 0.08), p=0.117	0.39, (0.03, 0.66), p=0.03	-	0.21, (-0.18, 0.54), p=0.284
**Calprotectin**	-0.22, (-0.54, 0.16), p=0.241	-0.22, (-0.55, 0.17), p=0.252	0.15, (-0.23, 0.49), p=0.432	0, (-0.36, 0.37), p=0.984	-0.21, (-0.54, 0.17), p=0.267	-0.08, (-0.44, 0.29), p=0.673	0.21, (-0.18, 0.54), p=0.284	-
